# Radiation-induced PGE_2_ sustains human glioma cell growth and survival through EGF signaling

**DOI:** 10.18632/oncotarget.3160

**Published:** 2015-02-25

**Authors:** Emeline Brocard, Kristell Oizel, Lisenn Lalier, Claire Pecqueur, François Paris, François M. Vallette, Lisa Oliver

**Affiliations:** ^1^ Centre de Recherche en Cancérologie Nantes Angers UMR INSERM 892, CNRS 6299, Université de Nantes, 44007 Nantes, France; ^2^ Université de Nantes, Faculté de Médecine, 44007 Nantes, France; ^3^ LaBCT, Institut de Cancérologie de l'Ouest, 44805 St Herblain cedex, France; ^4^ CHU de Nantes, 1 Place Alexis Ricordeau, 44093 Nantes, France

**Keywords:** Radiation, caspase, prostaglandin, glioblastoma, EGFR

## Abstract

*Glioblastoma Multiforme* (GBM) is the most common brain cancer in adults. Radiotherapy (RT) is the most effective post-operative treatment for the patients even though GBM is one of the most radio-resistant tumors. Dead or dying cells within the tumor are thought to promote resistance to treatment through mechanisms that are very poorly understood. We have evaluated the role of Prostaglandin E2 (PGE_2_), a versatile bioactive lipid, in GBM radio-resistance. We used an *in vitro* approach using 3D primary cultures derived from representative GBM patients. We show that irradiated glioma cells produced and released PGE_2_ in important quantities independently of the induction of cell death. We demonstrate that the addition of PGE_2_ enhances cell survival and proliferation though its ability to trans-activate the Epithelial Growth Factor receptor (EGFR) and to activate β-catenin. Indeed, PGE_2_ can substitute for EGF to promote primary cultures survival and growth *in vitro* and the effect is likely to occur though the Prostaglandin E2 receptor EP2.

## INTRODUCTION

Glioblastoma Multiforme (GBM) is the most common form of brain cancer in the adult and its prognosis remains poor. Standard therapy includes surgery followed by external beam radiotherapy (RT) and/or chemotherapy, principally temozolomide (TMZ), a DNA alkylating/methylating agent [1]. However, the effect of TMZ is beneficiary only to a subset of patients that do not express O-6-methylguanine-DNA methyltransferase (MGMT), a DNA repair enzyme [2]. GBM appear to be intrinsically extremely radio-resistant and this has been linked to the presence of cancer stem cells (CSC), which are efficient in repairing DNA damage [3]. Radiation resistance has also been linked to glioma heterogeneity [4], the lack of apoptosis [5] or activation of Wnt/β-catenin, Notch, EGF receptors and kinase signaling pathways [6–9].

Recent results have shown that RT induced a mitogenic signal that could originate from dying tumor and stromal cells [10]. Huang *et al*. [11] further demonstrated that after caspase activation dying cells released the bioactive lipid prostaglandin E2 (PGE_2_), which in turn was capable of triggering tumor repopulation. The ability of PGE_2_ to stimulate the proliferation of numerous types of cells has been shown both *in vivo* and *in vitro* and enzymes implicated in the synthesis of this prostanoid, such as cyclooxygenase 2 (Cox2), have been considered as a major target for anti-cancer therapies [10]. PGE_2_ is implicated in numerous mechanisms including the induction of cell migration to inflammation that can affect in cancer progression in various different ways. PGE_2_ has been shown to induce the synthesis of Bcl-2, a major anti-apoptotic protein in colon cancer and as such could directly control apoptosis [12]. On the other hand, we have shown that intracellular PGE_2_ triggers Bax, a pro-apoptotic protein, activation and as such would participate in the induction of apoptosis in both glioma and colon cancer [13–15]. These results suggest that PGE_2_ may play multiple and somewhat contradictory roles during cancer progression.

In the present study, we addressed the question of the role PGE_2_ on tumor progression and survival, using primary cultures derived from human GBM grown in 3D-cultures.

## RESULTS

### Irradiation of the human glioma cell line U251 induces the production of PGE_2_ without activation of caspase 3 or apoptosis

**Figure 1:**
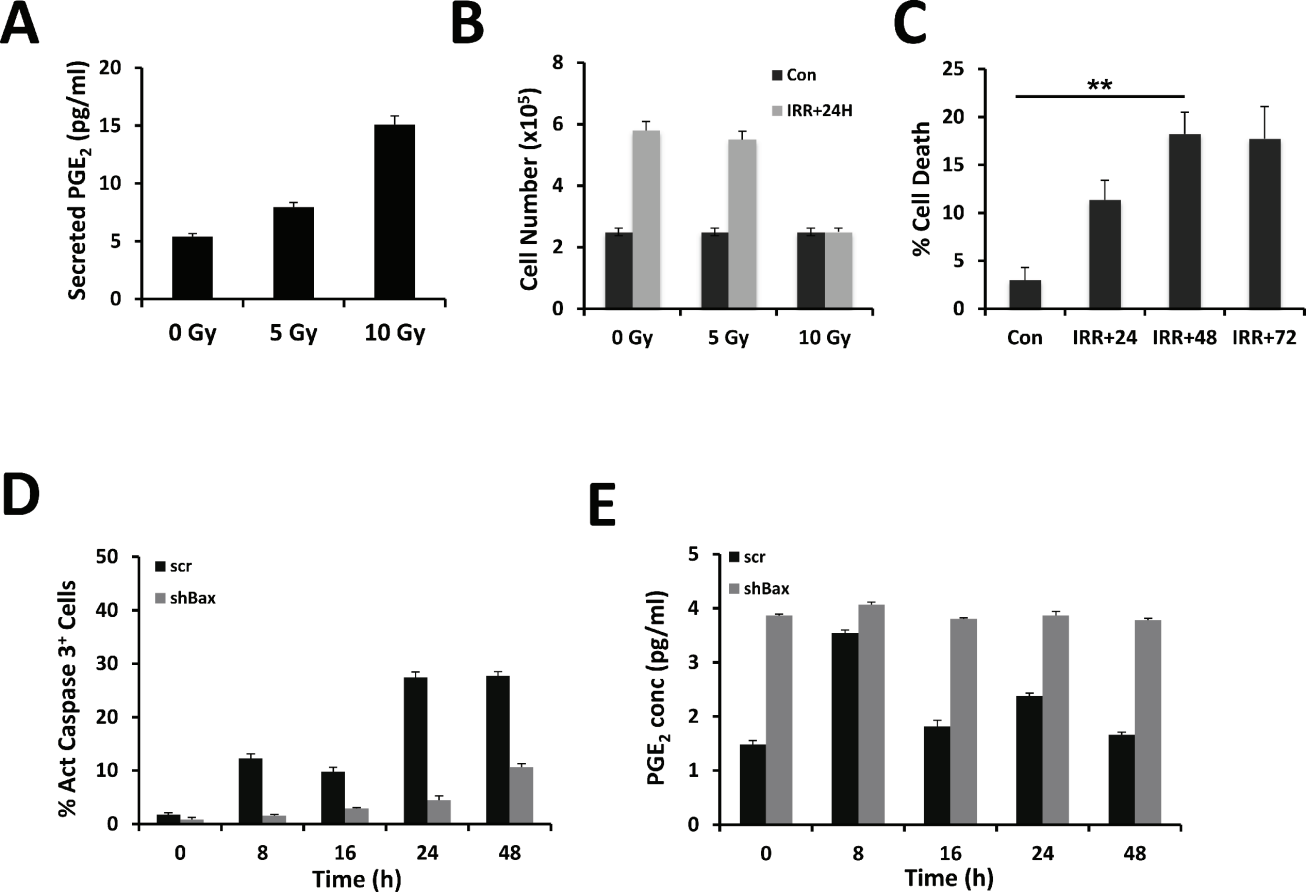
Release of PGE_2_ from γ-irradiated cells U251 cells were irradiated in serum-free medium at the indicated dose. PGE_2_ secretion was measured in supernatants after 24 h. The concentration of PGE_2_ (pg/ml) released from irradiated U251 cells was determined using an ELISA (see materials and methods) **(A)**. Cell viability was determined by trypan blue exclusion using the Countess automatic cell counter (Life Technologies), 24 h after irradiation of U251 cells **(B)**. Cell death was estimated as above at 24 h, 48 h and 72 h post-irradiation **(C)**. U251 cells were transduced with shRNA encoding viral particles [either encoding for a non-relevant shRNA (scr) or shRNA directed against Bax mRNA]. Cells were irradiated in serum-free medium at 10 Gy; harvested at the indicated time points, fixed and labeled with active caspase 3 antibody coupled to a fluorescent secondary antibody. The percentage of labeled cells was assessed by flow cytometry **(D)**. The corresponding PGE_2_ secretion was measured in supernatants during 48 h after irradiation at 5 Gy **(E)**. Please note that in the latter experiments, the secretion of PGE_2_ was decreased in scr-treated U251 compared to untreated cells (compare A and E).

The accumulation of PGE_2_ was measured 24 h after *γ*-irradiation of the human glioma cell line U251 at different intensities (i.e. 0, 5 and 10 Gy). As shown in Figure 1A, the amount of PGE_2_ found in the culture supernatant was proportional to the dose of radiation. Recent results have associated the induction of caspase activity in cancer cells to the production of PGE_2_ upon irradiation of cancer cells [11, 13–15]. We then assessed the viability of the cells under our conditions and found that only the high dose of irradiation (10 Gy) provoked a cell growth arrest and subsequent cell death after 48 h (Figure 1B, 1C). To determine whether the cell death was caspase 3 dependent, the number of active caspase 3 cells was quantified. As seen in Figure 1D, there was a close correlation between the percent of cell death and the percent of active caspase 3 cells. To evaluate the implication of caspase in the production of PGE_2_, we knocked down the expression of Bax, a central pro-apoptotic member of the BCL-2 family, in U251 cells. As illustrated in Figure 1D, we observed a significant decrease in the induction of caspase 3 activity in the absence of Bax after γ-irradiation at 10 Gy. However, quite surprisingly, the knock down of Bax appeared to promote the production of PGE_2_ in untreated U251 cells and the amount of PGE_2_ is maintained upon irradiation (Figure 1E), as previously observed in primary cultures of GBM [14]. Of note, the induction of PGE_2_ in control cells reached a peak 8 h after a 10 Gy irradiation to returned to normal at 16 h (Figure 1E).

We conclude from these results that PGE_2_ could be produced upon irradiation even in the absence of caspase activation.

### Irradiation, apoptosis and the expression of key members of the BCL-2 family in GBM primary cultures

**Figure 2:**
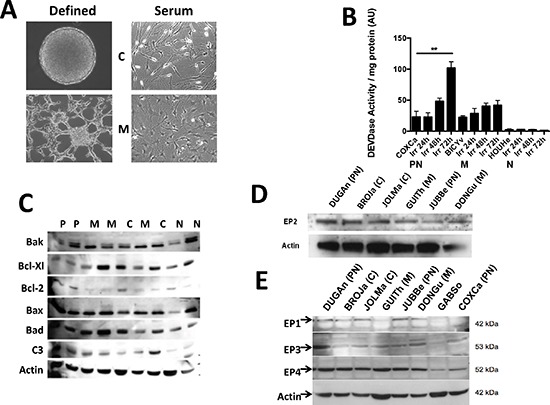
Characteristics of primary cultures derived from GBM patients Pictograph of primary cultures grown in defined (left) and serum-supplemented (right) media. Depending on the type of GBMs and/or culture conditions, cells can grow as spheres and/or as adherent cells (C = classical and M = mesenchymal subtypes) **(A)**. Caspase induction was measured in primary cultures after irradiation (10 Gy). The induction of caspase was observed in a single tumor. The cut-off for caspase 3 activation resulting in cell death was defined at 50 AU/mg protein **(B)**. The expression of key proteins of apoptosis was examined in primary cultures and illustrated in representative of the different subtype (PN = proneural, N = neural, C = classical and M = mesenchymal) **(C)**. The expression of the receptors of PGE_2_ (EP 1–4) was determined by western blots in the different representative primary cultures **(D, E)**.

To examine the biological consequence of the release of PGE_2_ by GBM, we used primary cultures derived from resected tumors. Cells dissociated from these tumors were capable of growing as neurospheres and/or as adherent cells in almost 80% of the cases. We were able to derive primary cultures, which correspond to proneural (young adults with a better prognosis but unresponsive to treatment), classical, neural and mesenchymal (older adults with a worse prognosis) classification of GBM corresponding to the molecular subtypes defined by Verhaak *et al*. [16] (Supplementary Figure S1 and Oliver et al. in preparation). Primary cultures in defined medium grow as spheres or as spheres and adherent cells whereas in serum complemented medium all primary cultures were adherent (Figure 2A). In all except one (15/16) primary culture we did not observed activation of caspase upon irradiation as similar low caspase 3 activities were observed in irradiated and untreated primary cultures. In Figure 2B, 3 primary cultures are given as an example (1/16 with high caspase activity, 3/16 with medium activity and 12/16 with low or no activity). Of note, the GBM cultures were able to undergo caspase dependent apoptosis as shown by their response to etoposide (Supplementary Figure S2). However, since a similar production of PGE_2_ was observed in all cells (data not shown), we conclude that it was independent of caspase activity and of the classes of GBM. Next we examined the expression of members of the BCL-2 family commonly found in GBM [17], in the different primary cultures. We observed an almost complete absence in the expression of Bcl-2 in 4/5 mesenchymal primary cultures while the other primary cultures expressed Bcl-2 in varying concentrations. The other proteins of the BCL-2 family such as Bcl-Xl, Bax, Bak or Bad were present in all the primary cultures at different levels (Figure 2C).

The expression of these proteins has been linked to PGE_2_ signaling pathways [12]. As shown in Figure 2D, EP2 was expressed in the four different types of GBM *in vitro* at similar level. Next, we examined the expression of the other PGE_2_ receptors *in vitro* (Figure 2E), we found that EP1, EP3 and EP4 receptors, which are also expressed in the brain [18], had a variable expression in primary cultures.

### Response of GBM primary cultures to PGE_2_

**Figure 3:**
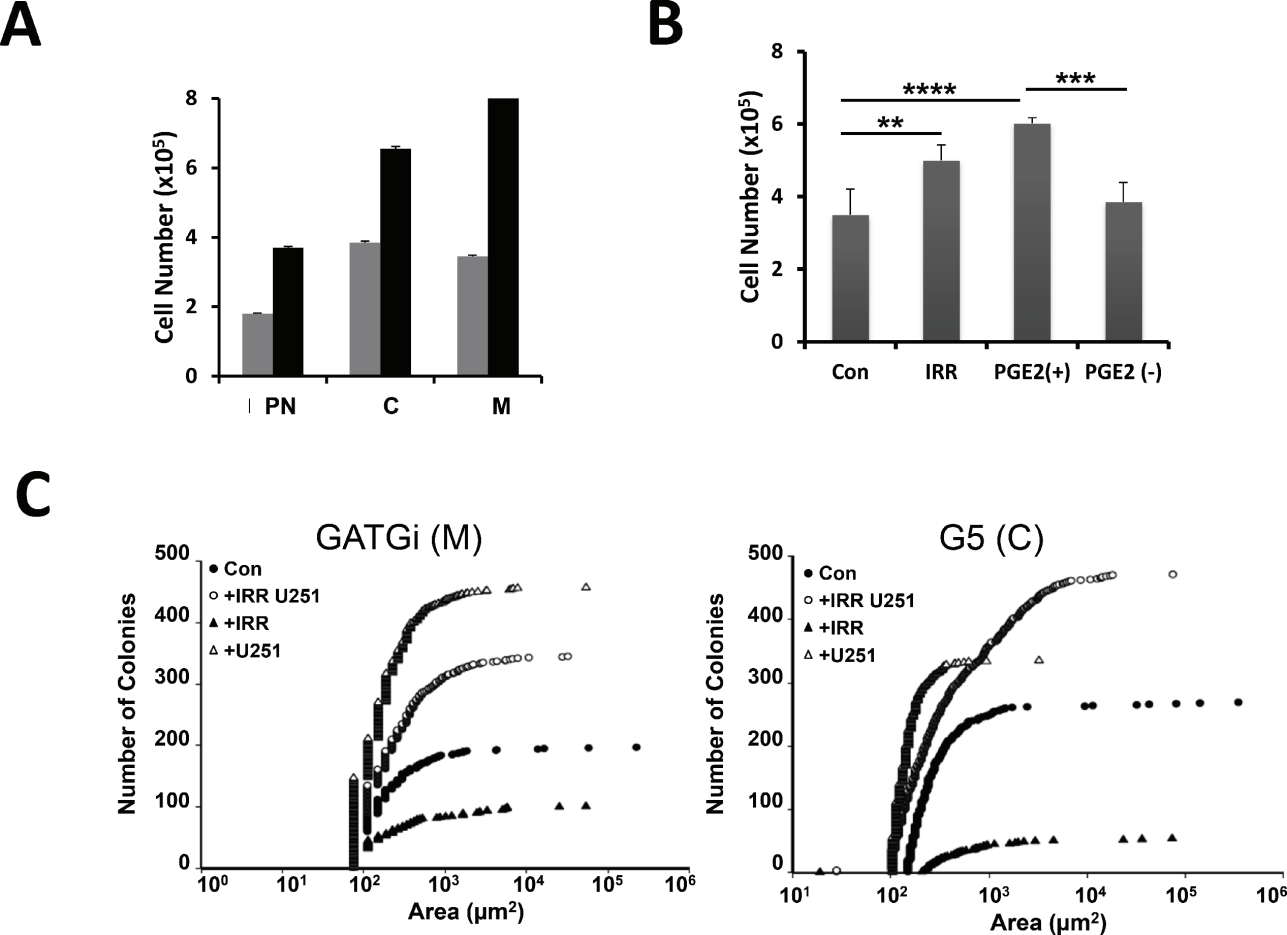
Effect of irradiation on GBM morphology and numbers *in vitro*; expression of PGE_2_ receptors in the primary cultures Primary cultures were either grown in defined medium (grey bars) or in 24 h-conditioned medium from U251 cells irradiated at 5 Gy (black bars) for 24 h, then cell number was determined using the Countess automatic cell counter. The data from representative cultures performed in triplicate are shown **(A)**. Primary culture was grown in the absence (con) or in the presence of supernatants from irradiated cells (IRR), supernatants immune-depleted in PGE_2_ (PGE2−) or in immune-depleted medium containing 10 μM PGE_2_ (PGE2+). Cell number was determined 24 h later as above **(B)**. (**p = 0,005; ***p = 0.0005; ****p < 0.0001). 3D co-culture in soft agar of primary cultures grown in the absence (con) or in the presence of irradiated U251 cells (+IRR). The +IRR cultures show smaller but much more colonies that the control cultures. GATGi and G5 are illustrating of mesenchymal (M) and classical (C) GBM sub-types **(C)**. Data are representative of 3 experiments done in duplicate.

Similar to U251, γ-irradiated primary cultures produced PGE_2_ although this production was heterogeneous and most of PGE_2_ remained associated to the spheres rather than released into the culture medium (data not shown). Primary GBM were cultivated in the presence of supernatant of irradiated cell culture media (ICCM). As shown in Figure 3A, cell proliferation was significantly increased upon incubation of primary cultures with ICCM. This effect was abolished after the immuno-depletion of PGE_2_. Note that the addition of PGE_2_ in immuno-depleted ICCM was sufficient to restore the effect on cell proliferation (Figure 3B). Next we used the 3D co-culture in soft agar system to determine the effects of the released PGE_2_ on the growth and survival of primary cultures. Under these experimental conditions U251 cells were irradiated with 5 Gy and 24 h later primary cultures were overlaid in soft agar and cultured for a further 3 weeks, as described in the methods section. Our first observation was the complete absence of large colonies in primary cultures overlaid over irradiated U251 cells as compared to control dishes (Figure 3C). However, the number of colonies in the co-cultures (primary cultures + irradiated U251 cells) was significantly increased. Next, we examined the expression of the different PGE_2_ receptors in the primary cultures: first the expression of EP2 as it is the most widely expressed prostaglandin receptor in the brain and it is functionally coupled to anti-apoptotic and protective functions in neurons and in secondary neurotoxicity during inflammation [18, 19].

**Figure 4:**
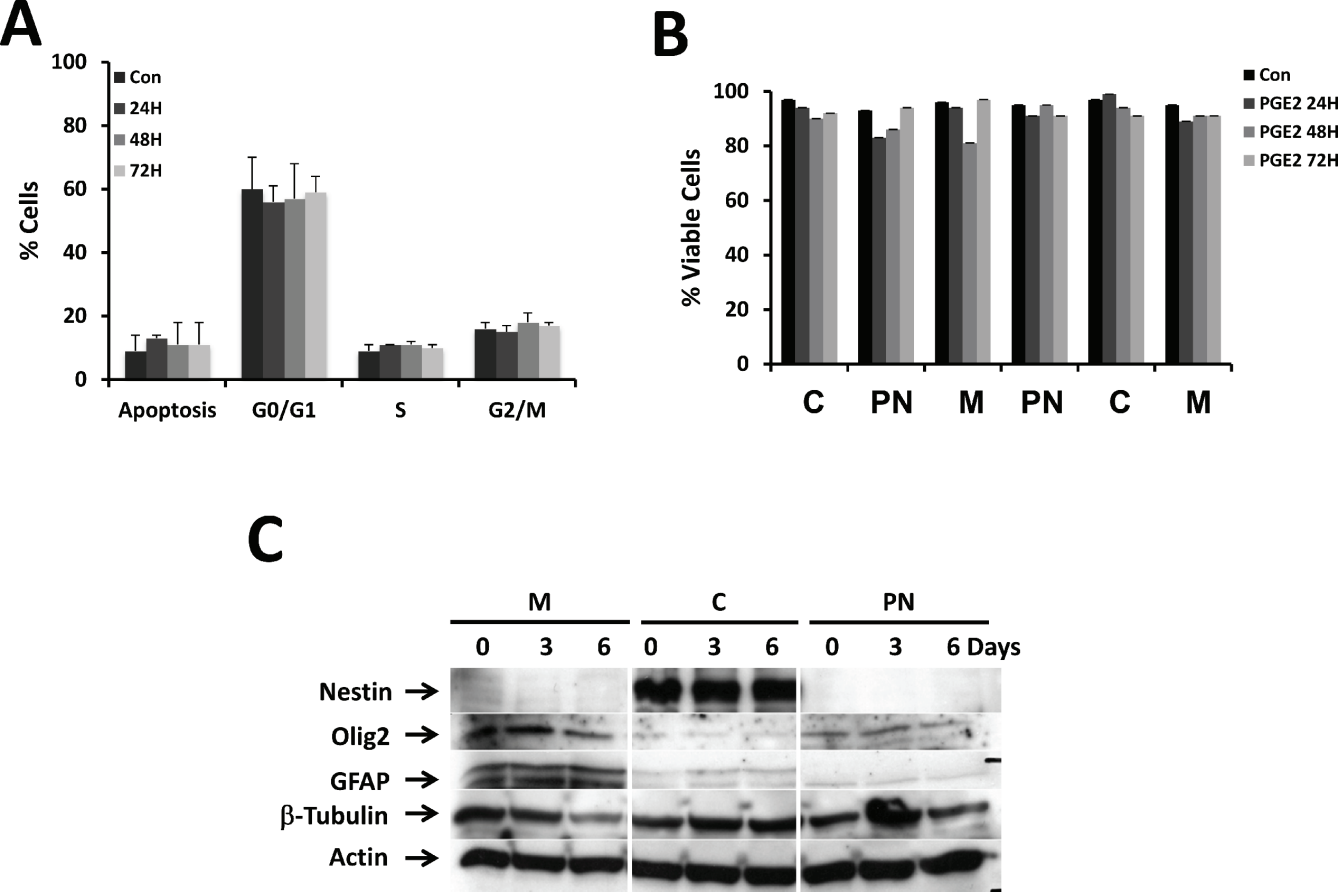
Effect of PGE_2_ on cell cycle, survival and expression of stemness markers The effect of PGE_2_ (10 μM) on cell cycle was analyzed together with the induction of apoptosis. As illustrated no effect on the addition of PGE_2_ on the different phases of the cycle **(A)** and cell viability **(B)** was observed over a period of 72 h. We also analyzed the effect of PGE_2_ on the expression of stemness markers under similar conditions and as illustrated in **(C)**, the expression of these markers was not affected by the treatment.

We did not observe any significant differences either on the cell cycle and apoptosis upon incubation with 10 μM PGE_2_ for 72 h between the different GBM cultures (Figure 4A) or on cell viability (Figure 4B).

It has been suggested that PGE_2_ liberated from dead or dying cells could regulate stem cells homeostasis and differentiation [20]. As our culture conditions support the survival and the proliferation of CSC [3], we examined if PGE_2_ could induce a change in the proportion of CSCs by analyzing the expression of stemness markers upon long-term exposure to the bioactive lipid. As shown in Figure 4C, the expression of stemness markers were markedly different in the different types of GBM primary cultures: some expressed markers for all types of neural cells (i.e. Olig2, GFAP and β-tubulin), and others expressing limited amount of neural markers (i.e. the neuronal β-tubulin) while others expressed high level of nestin and β-tubulin and low level of Olig2 and GFAP. In all cases incubation with PGE_2_ did not affect the nature and/or the level of these markers.

### Effect of PGE_2_ on the number and size of neurospheres in the presence or in the absence of EGF

**Figure 5:**
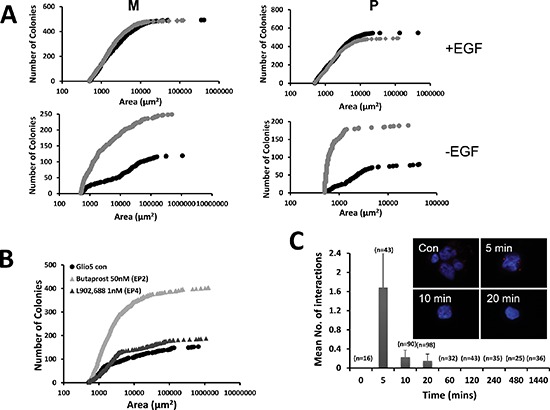
PGE_2_ and the EGF signaling pathway We examined the effect of PGE_2_ on both the amount of the sizes of spheres in the presence (top) or in the absence (bottom) of EGF. Primary cultures were grown in 3D cultures in the absence (black lines) or in the presence of 10 μM PGE_2_ for 3 weeks and the colonies formed were quantified using MetaMorph soft-ware **(A)** The data presented are representative of 3 independent experiments. Primary cultures were grown in soft agar in the absence or presence of agonist against EP2 (50 nM Butaprost) and EP4 (1 nM L902,688). After 3 weeks the number and size of neurospheres were determined **(B)**. PGE_2_ (10 μM) induces transactivation of EGFR. Proximity ligation *in situ* assay (P-LISA) adapted from the O-link protocol was used to monitor the phosphorylation of EGFR after addition of 10 mM PGE2 in the cells. Each red dot indicates a close proximity between EGFR (rabbit monoclonal) and pEGFR (mouse monoclonal). Graph represents the statistical analysis of the number of interactions versus time. The data presented are the mean of 2 experiments done in triplicate **(C)**.

PGE_2_ has been shown to trans-activate EGFR through its rapid phosphorylation [21, 22]. Since, GBM primary cultures are cultivated in the presence of EGF, we examined the effect of PGE_2_ in the absence or in the presence of this growth factor. We found that GBM primary cultures grew similarly in the presence or in the absence of PGE_2_ when EGF was present (Figure 5A). However, in absence of EGF, the addition of PGE_2_ increased both the size and the number of spheres, which reached in size, but not in numbers, a level similar to that observed in the presence of EGF (Figure 5A). Note that the addition of PGE_2_ alone did not have the same effect as PGE_2_ released from irradiated U251 cells. This could be due to the fact that the biolipid is very labile and has a very short half-life or to the presence of other factors. Possibly the continual release of PGE_2_ by dying cells has a more dramatic effect or the effect observed could be due to a combination of factors released.

Next we used agonists against EP2 and EP4 to determine the PGE_2_ receptor implicated in this effect. Results obtained in 3D cultures showed that Butaprost, an agonist of EP2 and not L902,688, an EP4 ligand, trigger a PGE2 like effect in the absence of EGF (Figure 5B). These results suggest that EGFR signaling could be implicated in the effect of PGE_2_ in primary cultures. EGFR was expressed in almost all primary cultures and basal phosphorylation monitored by immunoblot indicated that the receptor was phosphorylated under our conditions (Supplementary Figure S3). Incubation of primary cultures with PGE_2_ for 72 h did not show any significant activation of EGFR through increased phosphorylation (Supplementary Figure S3). Next we look at a short-term activation of EGFR by PGE_2_ using the OLINK technique [23]. As illustrated in Figure 5C, PGE_2_ induced a strong but transient phosphorylation of the receptor after 5 min incubation.

**Figure 6:**
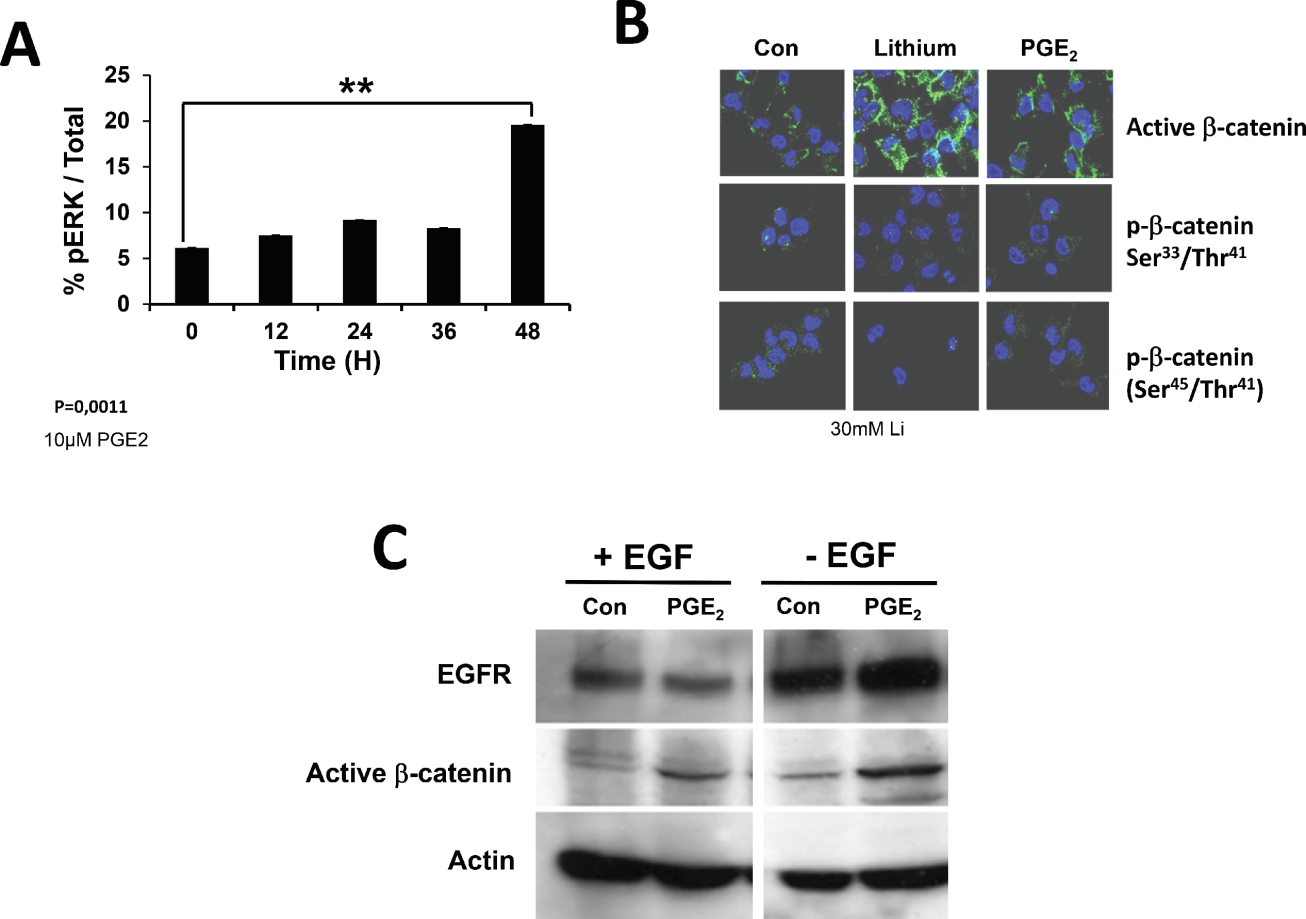
Activation of EGFR receptor monitored by ERK and β-catenin activation EGFR signaling pathway leads to the phosphorylation of ERK (pERK) and regulates β-catenin localization and stability (active β-catenin). We thus determine the percentage of pERK after incubation of primary cultures with PGE_2_ **(A)** and the intracellular localization of active b-catenin, using Lithium as a positive control **(B)**. We also analyzed the effect of PGE_2_ on EGFR and activeβ-catenin in the presence or in the absence of EGF **(C)**.

EGFR triggered the extracellular signal-regulated kinase (ERK)-mitogenic activated protein kinase (ERK/MAPK) signaling in many cells lines, an important component of radiation-induced hormesis [24] and glioma radio-resistance [25]. Primary cultures were treated with PGE_2_ and then the phosphorylation of ERK was quantified using a total phosphoERK1/2 ELISA. As shown in Figure 6A, the addition of PGE_2_ triggered a phosphorylation of ERK in primary cultures after 48 hours.

In colon cancer, PGE_2_ has been implicated in the induction of the Wnt/β-catenin pathway and as such to potentially play a critical role in carcinogenesis [26]. We analyze the activation of this pathway under our conditions by incubating primary cultures with 10 mM PGE_2_ for 48 h. Cells were than analyzed for β-catenin activation by immunohistochemistry (Figure 6B). The activation of β-catenin by PGE_2_ was compared to that induced by lithium and as shown in Figure 6B, both treatments led to the activation of β-catenin while on the other hand no effect on its phosphorylation was observed. We observed an increase in the induction of this activation and in the presence and in the absence of EGF, although the induction was more important in the latter case (Figure 6C).

## DISCUSSION

Glioma are resistant to chemo-therapy induced apoptosis [27]. Radio-therapy is the most effective therapy in GBM although these tumors remain largely refractory to the treatment. Mechanisms implicated in GBM radio-resistance are not well-known and unraveling them could provide new indications for better treatment strategies. Fractional/partial killing by radiation has been associated with a more rapid proliferation of the surviving cells and repopulation has been viewed over the longtime, as a major therapeutic challenge [28, 29]. This effect has been linked to the radiation-induced release of mitogenic factors by normal and/or cancer cells (30), the stimulation of radio-resistant tumor initiating cells [3, 31]. Recently, one potential mechanism, called “Phoenix Rising”, has been proposed to explain this effect [11, 20]. Huang et al. elegantly demonstrated that the proliferation signal could be generated by PGE_2_ produced upon activation of caspase 3, an enzyme essential for the completion of apoptosis, by radiotherapy [11]. We have shown that apoptosis is accompanied by an increase in the production of intracellular PGE_2_ in glioma and colon cancer but that this increase was upstream of the activation of caspase 3 [13–15]. We have observed that some GBM express high amounts of m-PGES1, the enzyme responsible for the synthesis of PGE_2_ from PGH, the product of COX2 [13]. Interestingly, the expression of m-PGES was associated with apoptosis and *in vitro* analyses indicated that this apoptosis was strictly Bax dependent [13–15]. We found that the production of PGE_2_ was triggered by most apoptotic inducers and that cells resistant to apoptosis accumulated and released abundant level of the lipid through MRP4, a PGE_2_ transporter both in glioma and colon cancer cells [13–15]. In the present work, we show that radiation can trigger PGE_2_ synthesis in glioma without inducing caspase activity. This PGE_2_ liberated participated in the survival and proliferation of surviving cancer cells by activating several pathways, including EGFR and β-catenin. Indeed, our results suggest that PGE_2_ can substitute for EGF to promote survival of irradiated cells. This observation is in agreement with numerous studies showing that accelerated repopulation during radiotherapy could be linked to the activation of EGFR and the subsequent activation of ERK/MAP kinase mitogenic pathways [28]. However, our results show also that PGE_2_ under our conditions is a pro-survival factor and that irradiated cells released pro-proliferative factors that remain to be identified.

PGE_2_ has also been shown to specifically reactivate the repopulation of normal cells by stimulating normal stem cells [32]. However, in our hands, we did not observed any change in the expression of stemness markers upon PGE_2_ treatment in glioma primary cultures regardless of the molecular sub-class of GBM and independently of the proportion of CD133^+^ cells.

The roles of PGE_2_ in non targeted and targeted effects of ionizing radiations, especially in the inflammatory context [33], or on stem cells homeostasis [32] are not fully understood. Recently, COX2 and PGE_2_ have been implicated in cancer progression but mixed results were obtained with COX2 inhibitors and radio-sensitization [34, 35]. Our results provide a molecular mechanism, by which PGE_2_ can sustain tumor growth and proliferation after tumor irradiation and support new alternative targets, such as EP2, to potentiate the effect of radiotherapy.

## MATERIALS AND METHODS

Unless stated otherwise, all cell culture material was obtained from Life Technologies (Cergy Pontoise, France) and chemicals were from Sigma-Aldrich (St. Louis, MO, USA).

### Cell culture

Human primary cultures were grown in defined medium (DMEM/HAM-F12, 2 mM L-glutamine, N2 and B27 supplement, 2 μg/ml heparin, 20 ng/ml EGF and 25 ng/ml bFGF, 100 U/ml penicillin and 100 μg/ml streptomycin and the U251-MG cell line was cultured in DMEM (4.5 g/L glucose), 10% fetal calf serum, 2 mM L-glutamine, 100 U/mL penicillin and 100 μg/mL streptomycin in an atmosphere of 5% CO_2_ and 95% humidity.

### 3D-culture

Primary GBM cells (2.5 × 10^3^) resuspended in 0.35% soft agar containing different compounds were layered on 0.5% agar. The soft agar layer was covered with media containing the compound to be tested. After 3 weeks, the cultures were scanned using a Leica DMI6000B and the Metamorph program. *γ*-irradiation was carried out in a Faxitron CP160 irradiator (Faxitron X-ray Corporation) at a dose rate of 1.48 Gy/min.

### ELISA

Quantity of phosphorylated and total protein was measured using the InstantOne ELISA kit (eBioscience) according to the manufacturer's instruction. Briefly, cells were lysed with 50 *μ*L lysis buffer and incubated 1 h at room temperature with 50 *μ*L antibody cocktail containing antibodies against total or phosphorylated forms and the peroxidase-labeled secondary antibodies. Colorimetric detection reagent (100 *μ*l) was added and the reaction read at 450 nm.

### Protein lysates, immunoblotting and caspase activity

Total proteins were extracted in (25 mM Tris-HCl, pH 7.6, 150 mM NaCl, 1% NP40, 1% Na-deoxycholate, 0.1% SDS) supplemented with protease inhibitors. Protein concentration was determined using BCA protein assay (Sigma). Proteins were separated by SDS-PAGE, transferred onto PVDF membrane (Millipore, St Quentin-Yvelines, France) and revealed with ECL (Millipore). Antibodies that recognize actin (Millipore), Bax (BD Pharmingen, San Jose, CA, USA), Bcl-2 (BD Pharmingen), EGFR and pEGFR (Cell Signaling Technology, Denvers, MA, USA), Bad (Cell Signaling), EP1–4 (Cayman, Ann Arbor, MI, USA), caspase 3 (Santa Cruz Biotech, Santa Cruz CA, USA), GFAP (Calbiochem, Darmstadt, Germany), nestin (Millipore, Temecula, CA, USA), olig2 (Abcam, Cambridge, UK) and β-tubulin (Sigma–Aldrich) were used. HRP-conjugated secondary antibodies were from BioRad. The ImageJ64 software was used to quantify Western blot bands. Caspase 3 activity was determined using the fluorogenic substrate Ac-DEVD-AMC, as described in [14]. Note, for all the assays the cut-off limit of caspase 3 activation that induces cell death was determined at > 50 AU/mg protein.

### PGE_2_ assay and immunodepletion

U251 cells (×10^5^) were seeded in 6 well plates. Complete medium was replaced by serum-free medium 24 h later and cells were γ-irradiated at the indicated intensity. Conditioned medium was recovered at indicated time points for PGE_2_ measurement. PGE_2_ assay was done according to the manufacturer's instructions (Amersham, GE Healthcare Europe, Velizy-Villacoublay, Fr). Cells were harvested and either assayed for cell viability or fixed and probed for active caspase 3 by flow cytometry (BD Pharmingen; 0.25 μg/10^6^ cells). When indicated, conditioned medium was depleted of PGE_2_ by filtration through PGE_2_ affinity column according to the manufacturer (Cayman).

### Olink

Cells were fixed with 4% paraformaldehyde in PBS for 15 min at room temperature then permeabilized with 0.1% SDS in PBS for 10 min at room temperature. Labeling was done according to the manufacturer's instructions (Duolink^®^ *In Situ*, Sigma–Aldrich). Fluorescence was visualized using the Axiovert 200 M microscope (Zeiss, Le Perq, France) with the apotome module (×63 objective and numerical aperture 1.4). Quantification was done using ImageJ.

The patients data have been kept confidential according to the recommendations of the National French Committee for Ethics and names of primary cultures correspond to codes.

## SUPPLEMENTARY FIGURES



## Abbreviations

CSC: cancer stem cells
Cyclooxygenase 2: Cox2
PGE_2_: Prostaglandin E2
EP2: Prostaglandin E receptor 2
EP4: prostaglandin E receptor 4
TMZ: temozolomide
RT: radiotherapy
