# Supplementary material for: Radiation-induced PGE2 sustains human glioma cell growth and survival through EGF signaling
Source: Oncotarget. 2015 Feb 25;6(9):6840–9. doi: 10.18632/oncotarget.3160 (PMC4466653; doi:10.18632/oncotarget.3160)
Supplement: Supplementary file 1 [file oncotarget-06-6840-s001.pdf]

SUPPLEMENTARY FIGURES

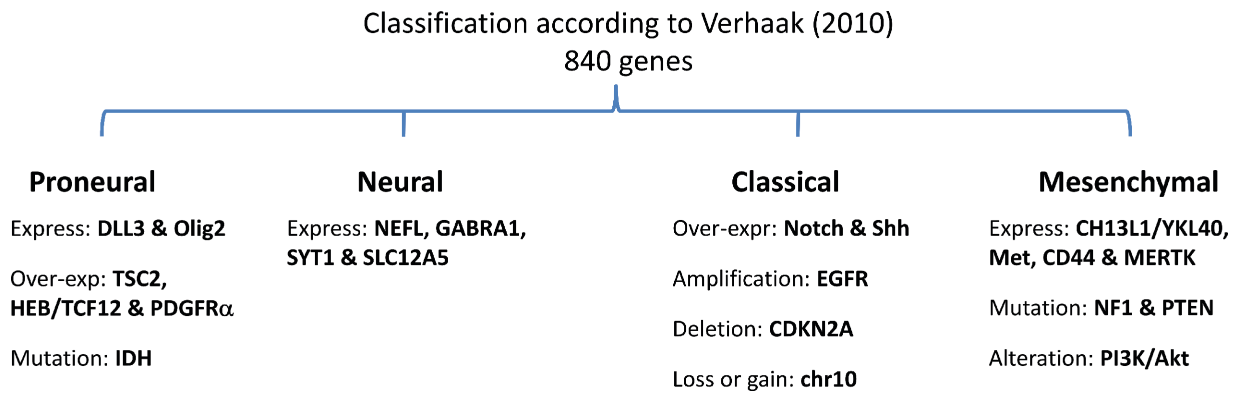

Supplementary Figure S1: Some specific molecular characterization of the different primary cultures according to Verhaak et al (ref 16).

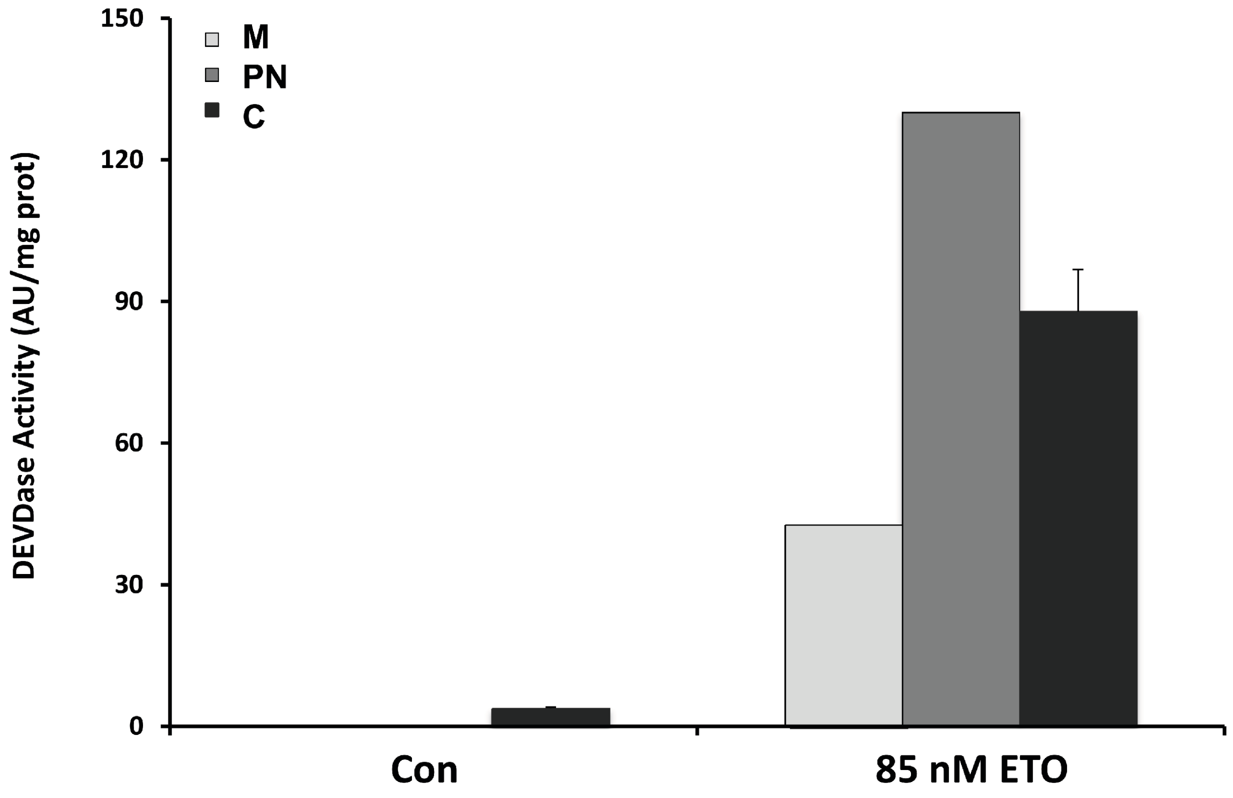

Supplementary Figure S2: Caspase dependent apoptosis can be induced in the GBM primary cultures by Etoposide (85 nM). Mesenchymal, proneural and classical primary cultures (*n* = 3) were treated with Etoposide (ETO) for 48 hrs before caspase 3 assays.

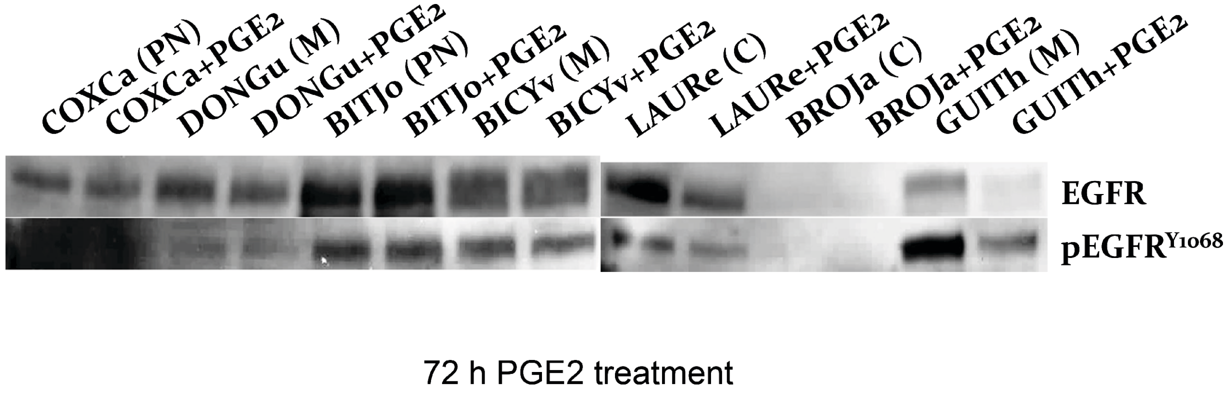

Supplementary Figure S3: Effect of PGE2 on EGFR expression and phosphorylation after 72 hrs exposure. Immunoblot with antiEGFR and anti PhosphoEGFR antibodies were performed as described in the "materials and methods" section.
